# Effects of Prevalent and Incident Chronic Kidney Disease on Cardiovascular Events in Patients with Atrial Fibrillation

**DOI:** 10.3390/jcm8081184

**Published:** 2019-08-07

**Authors:** Hsuan-Yu Lin, Chew-Teng Kor, Yao-Peng Hsieh, Ping-Fang Chiu

**Affiliations:** 1 Department of Internal Medicine, Changhua Christian Hospital, Changhua 50006, Taiwan; 2 Division of Nephrology, Department of Internal Medicine, Changhua Christian Hospital, Changhua 50006, Taiwan; 3 School of Medicine, Kaohsiung Medical University, Kaohsiung 80708, Taiwan; 4 School of Medicine, Chung Shan Medical University, Taichung 40201, Taiwan; 5 Department of Recreation and Holistic Wellness, MingDao University, Changhua 52345, Taiwan

**Keywords:** atrial fibrillation (AF), chronic kidney disease (CKD), mortality, myocardial infarction, stroke

## Abstract

Background: Chronic kidney disease (CKD) is a well-known complication of atrial fibrillation (AF) but how the incident CKD affects the clinical outcomes amongst AF patients is not clear. Methods: Our study data were retrieved from National Health Insurance Research Data for the period from 1996 to 2013. Incident AF patients were classified as non-CKD group (*n* = 7272), prevalent CKD group (*n* = 2104), and incident CKD group (*n* = 1507) based on administrative codes. Patients with prevalent CKD were those participants who already had CKD ahead of the index date of AF, whereas patients with incident CKD were those who developed CKD after the index date and the remaining patients were designated as non-CKD. Multivariate-adjusted time-dependent Cox models were conducted to estimate the associations of CKD status with the outcomes of interest, including heart failure (HF), acute myocardial infarction (AMI), stroke or systemic thromboembolism, all-cause mortality, and cardiovascular (CV) mortality, expressed as hazard ratio (HR) and 95% confidence interval (CI). Results: The mean age was 70.8 ± 13.3 years, and 55.4% of the studied population were men. In Cox models, the adjusted rate of HF, AMI, all-cause mortality, and CV mortality was greater in the prevalent and incident CKD groups, ranging from 1.31-fold to 4.28-fold, compared with non-CKD group. Notably, incident CKD was associated with higher rates of HF (HR, 1.8; 95% CI, 1.67–1.93), stroke or systemic thromboembolism (HR, 1.33; 95% CI, 1.22–1.45), AMI (HR, 1.46; 95% CI, 1.25–1.71), all-cause mortality (HR, 1.76; 95% CI, 1.68–1.85), and CV mortality (HR, 2.13; 95% CI, 1.92–2.36) compared with prevalent CKD. Conclusion: The presence of CKD was associated with higher risks of subsequent adverse clinical outcomes in patients with AF. Our study was even highlighted by the finding that incident CKD was linked to higher risks of outcome events compared with prevalent CKD.

## 1. Introduction

The prevalence of chronic kidney disease (CKD) has been escalating gradually owing to the aging population and higher comorbidity burden, such as diabetes mellitus and hypertension [1]. CKD is a well-recognized medical problem and carried a higher risk for death, hospitalization, and cardiovascular (CV) events [2]. CKD has been estimated to affect 13.1% of the United States population and medical costs for CKD is also vast as 33 billion US dollars was spent on the US ESRD program in 2010 [3,4]. However, work on development of effective and novel treatment models has failed to make significant advances in patient-centered clinical outcomes.

Similarly, the prevalence of atrial fibrillation (AF) is predicted to double by 2050 in the United States, with an estimated rate of five million incident cases globally [5,6]. Apart from higher medical costs, AF has a variety of CV effects with increased risks for heart failure (HF), acute myocardial infarction (AMI), and stroke [7,8]. CKD and AF are closely linked to each other through a common pathogenic mechanism. CKD is a risk factor of AF and vice versa [9,10,11]. A pooled analysis of three studies involving 467,000 patients with AF also showed the relative risk of CKD was 1.64 [12].

Over time, the health burden of CKD and AF is increasing, so a better understanding of the clinical consequences associated with incident CKD in patients with AF will have a major impact on the management of this high-risk population. In this study, we tested the hypothesis that AF patient with incident CKD would be associated with higher risk of all types of CV events compared with those who did not develop CKD. In addition, we also compared the event rates of CV diseases between AF patients with incident CKD and those with prevalent CKD.

## 2. Materials and Methods

### 2.1. Data Source

Taiwan National Health Insurance is a single-payer and compulsory scheme and has been launched since 1995 for all the residents in Taiwan with a coverage rate of >99%. The National Health Insurance Research Database (NHIRD) is developed and released by the Bureau of National Health Insurance (NHI) for scientific research. Our study data were retrieved from NHIRD for the period from 1996 to 2013. The database comprised detailed information on inpatient and outpatient claims, including demographics, diagnostic codes according to the International Classification of Diseases, Ninth Revision, Clinical Modification (ICD-9-CM), pharmacotherapy, the dates of hospital admission and discharge, and the date of death.

### 2.2. Study Cohort and Design

This study was conducted in compliance of the declaration of Helsinki and approved by the Institutional Review Board of Changhua Christian Hospital, Changhua, Taiwan. We were granted the waiver of informed consent for each participant because of the encryption of personal identification and the retrospective design of this study.

We used the following criteria to define a medical diagnosis: (1) the presence of a medical diagnosis by ICD-9-CM at the discharge diagnosis, or (2) the medical diagnosis was listed on the outpatient record for at least twice within one year and the time interval between the first and last medical diagnosis should be at least 90 days. First, we identified participants with incident AF for the period between 2000 and 2013 by excluding those with prevalent AF diagnosed from 1996 to 1999. The index date was determined on the date of incident AF onset. Second, we excluded those subjects who were <18 or >100 years old, followed <90 days or incomplete demographic data, or underwent dialysis or kidney transplant before the index date.

Study participants were further classified as having prevalent CKD, incident CKD or non-CKD based on the temporal relations between the date of incident AF and the date of CKD occurrence. Patients with prevalent CKD were those participants who already had CKD ahead of the index date of AF, whereas patients with incident CKD were those who developed CKD after the index date and the remaining patients were designated as non-CKD. All the outcomes were recorded during the entire follow-up period starting from the index date till 31 December 2013.

### 2.3. Study Outcomes and Relevant Confounding Variables

The endpoints of this study were all-cause mortality, CV mortality, acute myocardial infarction (AMI), heart failure (HF) and stroke or systemic thromboembolism which included ischemic stroke, pulmonary embolism, transient ischemic attack, and peripheral artery embolism. The events of AMI, HF, stroke or systemic thromboembolism were recorded if they were listed as the first diagnosis at discharge [13]. The causes of death were determined as either the main discharge diagnosis for in-hospital death or the first discharge diagnosis of the last hospitalization within three months prior to the death for death outside the hospital [14]. Apart from demographics, confounding factors for adjustment included baseline comorbidities, medications use, geographic locations, monthly incomes, the frequency of annual outpatient visits and CHA_2_DS_2_-VASc score [15]. The ICD-9-CM codes used to identify comorbidities and the cause of death were listed in Table S1.

### 2.4. Statistical Analyses

Frequency (percentage) or mean ± standard deviation (SD) was used to show the data distribution, as appropriate. All the baseline characteristics, including demographic data, comorbidities, pharmacotherapy, CHA_2_DS_2_-VASc score, and annual outpatient visits between three groups were compared using Chi-square test, or one-way analysis of variance (ANOVA), as appropriate. The 1000 person-years incidences of the study outcomes with 95% confidence interval (CI) were calculated for the three CKD groups. Because CKD was a time-update exposure, crude cumulative incidence rates of study outcomes for three groups were compared using Simon and Makuch method that is an alternative for Kaplan-Meier estimate and takes into account the change in an individual’s covariate status over time.

Propensity scores have been widely used by a substantial of work to match, stratify and weight the samples from the study cohorts to eliminate or reduce confounding via achieving similar distributions of observed pretreatment covariates between the control and treatment groups. To reduce the confounding effects due to the imbalances in baseline characteristics distribution, generalized boosted regression model was used to estimate the propensity scores for the three groups based on all the baseline covariates. Given the concern over the three, rather than two, treatment (exposure) groups in our study, we next performed the estimated propensity of CKD groups from generalized boosted regression to generate an inverse probability of group weighting (IPW) to fit to control for the imbalances on observed variables [16,17]. The generalized boosted regression procedure was implemented using the twang package for R (twang: Toolkit for Weighting and Analysis of Nonequivalent Groups; R package version R i386 3.5.2). The balance of covariate distribution before and after IPW was evaluated using standardized differences. A standardized difference of <10% indicated the balance on their propensity score weighted distribution of covariates between different groups.

Multivariable Cox proportional hazards regression models were performed to examine the association between CKD status and risk of our outcome events. In light of immortal time bias for the incident CKD group, competing risk of death and the three CKD groups in our study, IPW-adjusted time-dependent cause-specific Cox models were performed to compare the risk of study endpoints by the three CKD groups, which was shown by hazard ratios (HRs) and 95% confidence interval (CI). In our statistical analyses, CKD was treated as a time-updated exposure. Thus, for the incident CKD group, they contributed person-time to the non-CKD group before being diagnosed with CKD and would contribute person-time to the incident CKD group after being diagnosed with CKD.

We repeated our analyses to examine the association of CKD status with study outcomes in various subgroups, stratified by age (< or ≥65 years), gender, CHA_2_DS_2_-VASc score (< or ≥3) and the use of renin-angiotensin system inhibitors. In addition, we also conducted a serial of sensitivity tests. First, we repeated our analyses after excluding AF patients with valvular heart disease or hyperthyroidism. Second, we re-defined the CKD diagnosis by ICD-9 code 585. Third, the primary results were adjusted for the propensity score to test the robustness. Finally, we restricted the study population to AF patients with follow-up of at least 1 year. All the sensitivity analyses were performed by treating antiplatelet and anticoagulant agents as time-dependent variables to determine whether those treatments attenuate the observed associations. Furthermore, the temporal difference of reporting CKD is a significant issue in our study and misclassification of prevalent and incident CKD was possible. We re-classified those who developed CKD within 30, 90 and 180 days of AF diagnosis as having prevalent CKD to test the robustness of our study. R language and SPSS statistical software, version 20.0 (SAS Institute Inc., Cary, NC, USA) were used to analyze the data and statistical significance was set at a two-tailed *p* value < 0.05.

## 3. Results

### 3.1. Characteristics of Patients

**Figure 1:**
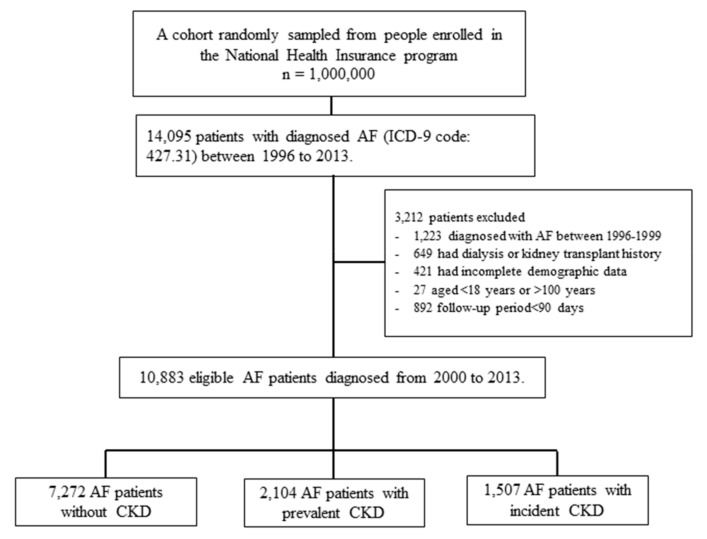
Flowchart of patient selection processes for incident atrial fibrillation (AF) patients with non-chronic kidney disease (CKD), prevalent CKD and incident CKD.

During 2000 to 2013, a total of 10,883 participants were eligible for the study and they were classified as non-CKD group (*n* = 7272), prevalent CKD group (*n* = 2104) and incident CKD group (*n* = 1507) (Figure 1). The distributions of baseline demographic characteristics, comorbidities, and pharmacotherapy for the study cohort by CKD status were shown in Table 1. The mean time of follow-up was 4.37 ± 3.50 years for the entire cohort. The mean age was 70.8 years (SD, 13.3), and 55.4% (*n* = 6029) of the studied population were men. There were marked differences in the distribution of covariates among the three CKD groups. Patients with CKD (prevalent or incident) were more likely to be older, men, have claims for chronic comorbidities and higher CHA_2_DS_2_-VASc scores compared with those without CKD. After IPW using propensity, all the covariates were distributed without significant difference based on the maximum standardization differences of less than 0.1 that means negligible difference.

### 3.2. Rates of Study Outcomes by CKD Status

**Figure 2:**
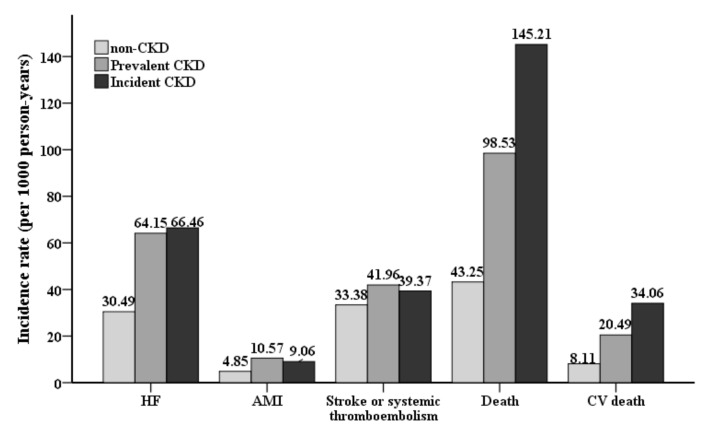
Cumulative incidence rate of study outcomes between prevalent CKD, non-CKD and incident CKD groups.

During the follow-up period of 14 years, the crude rate of HF hospitalization was about two-fold higher in patients with CKD (prevalent or incident) compared with those without CKD (Figure 2). Similar trends were observed regarding all-cause mortality, CV mortality, AMI, ischemic stroke or systemic thromboembolism. Intriguingly, the unadjusted rates of all-cause mortality and CV mortality were higher in the incident CKD group than those in the prevalent CKD group.

### 3.3. Association of CKD Status with the Risk of Subsequent Outcome Events 

The cumulative incidence of our study outcomes was plotted for the CKD groups in Figures S1–S5. In IPW-adjusted cause-specific and time-dependent multivariate Cox analyses, incident CKD was significantly associated with 2.36-fold higher rate of HF and prevalent CKD was associated with 1.31-fold higher rate of HF compared with non-CKD (Table 2). Furthermore, the adjusted rate of AMI, all-cause mortality and CV mortality was greater in the prevalent and incident CKD groups, ranging from 1.59-fold to 4.28-fold, compared with non-CKD group. Compared with the non-CKD group, the adjusted HR of stroke or systemic thromboembolism for incident CKD group was 28% higher (HR, 1.28; 95% CI, 1.18–1.38), while there was no significant difference between prevalent CKD and non-CKD groups. Notably, incident CKD was associated with higher rates of HF (HR, 1.8; 95% CI, 1.67–1.93), stroke or systemic thromboembolism (HR, 1.33; 95% CI, 1.22–1.45), AMI (HR, 1.46; 95% CI, 1.25–1.71) all-cause mortality (HR, 1.76; 95% CI, 1.68–1.85) and CV mortality (HR, 2.13; 95% CI, 1.92–2.36) compared with prevalent CKD.

### 3.4. Subgroup Analyses 

We tested for the potential interactions with some covariates by doing subgroup analyses stratified by age (< or ≥65 years), gender, CHA_2_DS_2_-VASc score (< or ≥3) and renin-angiotensin system inhibitors (Table 3). Consistent results were obtained showing higher risks of HF, AMI, all-cause mortality and CV mortality in the prevalent and incident CKD group compared with non-CKD group across most of these subgroups. In terms of stroke or systemic thromboembolism, there was no distinct difference between prevalent and non-CKD but no interaction test was statistically significant.

### 3.5. Sensitivity Analyses 

The results of the sensitivity analyses were shown in Table 4. The consistent results supported the main findings in Table 2, demonstrating that CKD was associated with higher risk of subsequent outcomes under investigation. The associations of different CKD status with clinical outcomes were consistent with the primary results after re-classifying those who developed CKD within 30, 90 and 180 days of AF diagnosis as having prevalent CKD (Tables S2 and S3).

## 4. Discussion

Using the nationwide representative healthcare data, we reported for the first time the association of CKD status (prevalent, incident or without) with the risk of various diverse clinical outcomes over a long observation period, up to 14 years, in a cohort of AF patients. Our findings are novel in that, (i) compared with non-CKD, both incident and prevalent CKD were associated with a 1.31- to 4.28-fold increased risk of HF, AMI, all-cause death, and CV death after accounting for a broad range of crucial confounders that mediate the occurrence of CKD, AF and adverse events; (ii) regarding stroke or systemic thromboembolism, the higher risk was only seen in incident CKD but not in prevalent CKD; (iii) incident CKD had even higher risk of all the adverse events compared with prevalent CKD. The independent effects of CKD remained consistent and robust while treating medications (antiplatelet and anticoagulant agents) as time-dependent variables and performing stratified and sensitivity analyses.

The prevalence rate of CKD in patients with AF varies a lot in different reports. In the AnTicoagulation and Risk Factors in Atrial Fibrillation (ATRIA) study, 43% patients had an estimated glomerular filtration rate (eGFR) at baseline < 0 mL/min/1.73 m^2^, whereas 16% and 29% was reported to have eGFR < 50 mL/min/1.73 m^2^ in two other randomized clinical trials [18,19,20], respectively. In light of the independent and adverse impact of CKD on cardiovascular outcomes, CKD undoubtedly amplifies the AF related complications, such as stroke and mortality. For example, both proteinuria and CKD (eGFR < 60 mL/min/1.73 m^2^) at baseline were associated with an increased risk of stroke after adjustment for known risk factors in patients with AF [18]. CKD was also reported to independently predict cardiovascular event (cardiac events and ischemic stroke) and all-cause mortality in patients with nonvalvular AF [21]. In a large, international AF trial of patients with nonvalvular AF at moderate to high risk of stroke, renal dysfunction has been proposed to be included in the stroke risk stratification scores [22].

Previous studies have confirmed the association between prevalent CKD and adverse CV events amongst AF population; however, evidence for the role of the incident CKD is limited so far. Guo et al. investigated the dynamic change in renal function and the risk of clinical outcomes in an AF cohort of 617 patients over 2 years [23]. Compared with patients with eGFR > 60 mL/min/1.73 m^2^ at six months of follow up, those with eGFR ≤ 60 mL/min/1.73 m^2^ were at significantly higher risks of death and stroke or death. The same association was found when the time period extended to 12 months. An absolute decrease and a relative decline in eGFR also independently predicted the risk for death and stroke or death. In our study, we also evaluated the impact of incident CKD on clinical events compared with non-CKD in a large AF cohort. We reported similar results showing the associations of incident CKD with subsequent stroke and mortality. The key differences included longer median follow-up time, the use of time-updated CKD status and focuses on other outcomes (such as HF and AMI) in our study. Therefore, we provided more robust evidence supporting the association between incident CKD and adverse CV events in adults with AF. In addition, we also found for the first time that incident CKD had even higher risks of HF, stroke, mortality, CV mortality and AMI compared with prevalent CKD. AF patients with concurrent CKD when they were diagnosed with AF undoubtedly will get more medical attention initially for these two comorbidities. Based on our findings that incident CKD carries the highest risks of all CV events compared with non-CKD and prevalent CKD. Patients with AF who do not have CKD initially should be regularly monitored for renal function to identify incident CKD earlier. Whether timely delivery of targeted therapeutic treatments to incident CKD patients can improve their clinical outcomes requires further large-scale prospective studies.

CKD and CV disease share the same pathophysiological mechanisms and common risk factors, such as diabetes and hypertension. Our study population was patients with AF who were at increased risks of all-cause mortality, stroke, cardiovascular mortality, heart failure, chronic kidney disease, ischemic heart disease and major cardiovascular events [12]. The mechanisms linking AF with non-stroke complications are so far unclear. There are several possible reasons to explain our findings. First, CKD is associated with a high prevalence of traditional CV risk factors, such as older age, diabetes, hypertension, and hyperlipidemia, all of which could promote the development of subsequent CV events [24]. This explanation seems unlikely because in the present study incident CKD remained to be associated with higher rates of CV events even after adjustment for those risk factors. Second, a broad spectrum of CKD-specific factors has been identified to be novel CV risk factors, including higher levels of inflammation, oxidative stress, endothelial dysfunction, asymmetric dimethylarginine and prothrombogenic factors [25,26,27]. Furthermore, the putative mechanisms by which CKD patients were at high risk of cerebrovascular disease included loss of calcification inhibitor, hyperphosphatemia, increased blood pressure variability, platelet dysfunction, and translocation of gut-derived bacterial toxins into the systemic circulation [28]. Third, a reduced GFR may simply represent an intermediary for vascular damage associated with future CV events. Renal arteriosclerosis has been reported to be closely related to systemic atherosclerosis [29]. Furthermore, the activation of renin-angiotensin-aldosterone system (RAAS) is also the most probable link between AF and CKD, leading to a variety of adverse clinical outcomes. RAAS is one of the main cause of CKD progression and its blockade reduces proteinuria and slows the decline in kidney function [30]. On the other hand, the RAAS is also thought to play a key role in the development of AF and its inhibition appears to reduce the risk of AF events [31]. Therefore, the activation of RAAS may be the putative mechanism that the coexistence of CKD and AF leads to more clinical events than AF alone.

Our study has several strengths. Taiwan NHI scheme is a single-payer and compulsory national insurance system that covers all medical care for all the beneficiaries so they only pay a relatively low copayment. Nearly all the residents in Taiwan were enrolled in NHI system so they had free access to health care, thus mitigating the reimbursement or selection bias. We were able to collect a wide range of comorbidities that may mediate CKD, AF and our study events through validated diagnostic codes in both the inpatient and outpatient departments. We studied the association of CKD (prevalent or incident) with the subsequent risk of clinical outcomes using cause-specific Cox model which accounted for the competing risk of death and using IPW estimation with propensity scores which considered the imbalance of covariate distribution for three or more groups. Moreover, a time-dependent model was used to classify incident CKD as a time-updated exposure given patients with incident CKD contributed time to the non-CKD group prior to CKD diagnosis. Thus, we believe that our findings were more reliable and valid compared with that using traditional Cox regression models.

Our study also had several limitations. First, the number of patients in the incident and prevalent CKD group is relatively small compared with non-CKD group that a small number of study events may affect the statistical power to detect significant differences in outcomes. Since incident CKD conferred a significantly higher risk of outcomes in our study, studies with more patients with incident CKD would further increase the magnitude of associations with clinical outcomes. Second, we were not able to exclude the presence of residual confounding factors, such as those novel CV risk factors like oxidative stress and inflammation. Instead, we adjusted for a variety of comorbidities which were the ultimate consequences of those risk factors. Third, the NHIRD lacks imaging and laboratory information, such as serum creatinine levels and proteinuria. Therefore, misclassification of CKD is a great concern. However, previous studies have evaluated the validity of CKD diagnosis from administrative codes and showed this approach is highly accurate to ascertain CKD status [32,33]. Also, numerous high-quality papers have been published using Taiwan NHIRD [14,34]. Finally, the causality cannot be inferred from the present study due to the retrospective design. Since CKD is a chronic disease that develops gradually, a degree of this entity could have been present before being diagnosed by administrative codes. However, consistent results while classifying those diagnosed with CKD within 30, 90 and 180 days of AF diagnosis further increased the robustness of our findings.

## 5. Conclusions

In conclusion, we observe that the presence of CKD, either prevalent or incident, was notably associated with higher risks of subsequent adverse clinical outcomes among a well-characterized cohort with AF. The associations were independent of various confounders and remained consistent in sensitivity tests and across most of the stratified analyses. Our study was even highlighted by the finding that incident CKD was linked to higher risks of outcome events compared with prevalent CKD. Frequent monitoring of renal function is advised for early detection of CKD occurrence with subsequent adoption of interventional therapy to reduce or mitigate the adverse CV outcomes.

## Figures and Tables

**Table 1:** Baseline characteristics of study population between the CKD groups.

	AF Cohort				Maximum Standardization Difference between Groups	
	Non-CKD	Prevalent CKD	Incident CKD	*p*-Value	Before IPW ^a^ (%)	After IPW ^a^ (%)
Sample size	7272	2104	1507			
Age, years	69.14 ± 13.85	75.37 ± 10.42	72.41 ± 11.01	<0.001	0.475	0.065
Gender, Male	3915 (53.84%)	1237 (58.79%)	877 (58.2%)	<0.001	0.100	0.037
Monthly income, NTD	13,373.07 ± 14398.08	10,225.88 ± 10,842.82	10,383.04 ± 11,327.26	<0.001	0.234	0.059
Geographic location						
Northern	3540 (48.68%)	954 (45.34%)	669 (44.39%)	0.001	0.086	0.027
Middle	1326 (18.23%)	390 (18.54%)	307 (20.37%)	0.152	0.054	0.024
Southern	2164 (29.76%)	691 (32.84%)	474 (31.45%)	0.019	0.066	0.013
Eastern	242 (3.33%)	69 (3.28%)	57 (3.78%)	0.647	0.027	0.026
Comorbidities within 1 year before the index date						
Ischemic heart disease	2587 (35.57%)	931 (44.25%)	679 (45.06%)	<0.001	0.195	0.036
Chronic obstructive pulmonary disease	1185 (16.3%)	524 (24.9%)	329 (21.83%)	<0.001	0.221	0.033
Cancer	463 (6.37%)	145 (6.89%)	98 (6.5%)	0.690	0.021	0.006
Liver Cirrhosis	100 (1.38%)	42 (2%)	36 (2.39%)	0.006	0.080	0.015
Dementia	233 (3.2%)	149 (7.08%)	54 (3.58%)	<0.001	0.198	0.005
Rheumatoid disease	96 (1.32%)	57 (2.71%)	25 (1.66%)	<0.001	0.110	0.022
CHA_2_DS_2_-VASc score	2.97 ± 1.78	3.93 ± 1.66	3.46 ± 1.66	<0.001	0.538	0.065
Long-term medication use						
Anti-hypertensive drugs						
renin-angiotensin system inhibitors	2458 (33.8%)	1210 (57.51%)	631 (41.87%)	<0.001	0.485	0.053
beta-blocker	2399 (32.99%)	982 (46.67%)	549 (36.43%)	<0.001	0.285	0.013
diuretics	1840 (25.3%)	1034 (49.14%)	509 (33.78%)	<0.001	0.515	0.038
Statin	985 (13.55%)	574 (27.28%)	228 (15.13%)	<0.001	0.371	0.033
NSAIDs	983 (13.52%)	504 (23.95%)	195 (12.94%)	<0.001	0.305	0.040
Pentoxifylline	252 (3.47%)	208 (9.89%)	51 (3.38%)	<0.001	0.307	0.056
ESA	0 (0%)	11 (0.52%)	0 (0%)	<0.001	0.165	0.038
Aspirin/ clopidogrel	2213 (30.43%)	981 (46.63%)	545 (36.16%)	<0.001	0.341	0.012
Warfarin	464 (6.38%)	145 (6.89%)	89 (5.91%)	0.482	0.040	0.046
NOACs	21 (0.29%)	2 (0.10%)	0 (0%)	0.033	0.063	0.049
Outpatient visit within 1 year before the index date	26.08 ± 19.47	34.6 ± 21.39	30.67 ± 21.24	<0.001	0.417	0.025

Values are expressed as mean ± SD or number (%). ^a^ Inverse probability of group-weighting (IPW) was estimated by the propensity of group from the generalized boosted regression. CKD, chronic kidney disease; AF, atrial fibrillation; NTD, New Taiwan Dollar; NSAID, non-steroidal anti-inflammatory drug; ESA, erythropoiesis-stimulating agents; NOAC, non-vitamin K oral anticoagulants.

**Table 2:** Risks for heart failure, stroke or systemic thromboembolism, acute myocardial infarction and mortality among patients with AF by CKD status.

Outcome	Event	IR (95% CI)	Weighted Time-Dependent Cox Model					
			cHR (95% CI)	*p*-Value	aHR (95% CI)	*p*-Value	aHR (95% CI)	*p*-Value
Heart failure								
Non-CKD	1174	30.49 (28.75–32.23)	1		1		0.76 (0.72–0.81)	<0.0001
Prevalent CKD	471	64.15 (58.35–69.94)	1.34 (1.27−1.42)	<0.0001	1.31 (1.24−1.39)	<0.0001	1	
Incident CKD	200	66.46 (57.25–75.67)	2.61 (2.44−2.80)	<0.0001	2.36 (2.20−2.53)	<0.0001	1.8 (1.67–1.93)	<0.0001
Stroke or systemic thromboembolism								
Non-CKD	1264	33.38 (31.54–35.22)	1		1		1.04 (0.97–1.11)	0.2308
Prevalent CKD	316	41.96 (37.33–46.59)	0.97 (0.91−1.04)	0.413	0.96 (0.90−1.03)	0.2539	1	
Incident CKD	150	39.37 (33.07–45.67)	1.39 (1.28−1.50)	<0.0001	1.28 (1.18−1.38)	<0.0001	1.33 (1.22–1.45)	<0.0001
Acute myocardial infarction								
Non-CKD	198	4.85 (4.17–5.52)	1		1		0.63 (0.55–0.72)	<0.0001
Prevalent CKD	87	10.57 (8.35–12.79)	1.65 (1.44–1.88)	<0.0001	1.59 (1.39–1.81)	<0.0001	1	
Incident CKD	39	9.06 (6.22–11.91)	2.61 (2.23–3.04)	<0.0001	2.32 (1.98–2.71)	<0.0001	1.46 (1.25–1.71)	<0.0001
All-cause mortality								
Non-CKD	1787	43.25 (41.24–45.25)	1		1		0.53 (0.5–0.56)	<0.0001
Prevalent CKD	827	98.53 (91.82–105.25)	1.98(1.89−2.08)	<0.0001	1.90 (1.81−2.00)	<0.0001	1	
Incident CKD	648	145.21 (134.03–156.39)	4.05(3.85−4.25)	<0.0001	3.37 (3.20−3.54)	<0.0001	1.76 (1.68–1.85)	<0.0001
Cardiovascular death								
Non-CKD	335	8.11 (7.24–8.98)	1		1		0.5 (0.45–0.56)	<0.0001
Prevalent CKD	172	20.49 (17.43–23.56)	2.10 (1.88−2.35)	<0.0001	2.01 (1.79−2.25)	<0.0001	1	
Incident CKD	152	34.06 (28.65–39.48)	5.08 (4.55−5.67)	<0.0001	4.28 (3.83−4.78)	<0.0001	2.13 (1.92–2.36)	<0.0001

CI, confidence interval; HR, hazard ratio; IR, incidence rate (per 1000 person-years); CKD, chronic kidney disease; AF, atrial fibrillation. aHR was calculated from adjustment for all variables in Table 1.

**Table 3:** Adjusted associations of CKD status with risk of clinical outcomes stratified by sex, age, CHA_2_DS_2_-VASc score and medication use.

Subgroup	Heart Failure		Stroke or Systemic Thromboembolism		Acute Myocardial Infarction		All-Cause Mortality		Cardiovascular Mortality	
	Prevalent CKD vs. Non-CKD	Incident CKD vs. Non-CKD	Prevalent CKD vs. Non-CKD	Incident CKD vs. Non-CKD	Prevalent CKD vs. Non-CKD	Incident CKD vs. Non-CKD	Prevalent CKD vs. Non-CKD	Incident CKD vs. Non-CKD	Prevalent CKD vs. Non-CKD	Incident CKD vs. Non-CKD
**Age**										
Age < 65	1.36 (1.20–1.54)	1.67 (1.42–1.96)	0.83 (0.58–1.18)	1.41 (0.97–2.05)	2.14 (1.64–2.80)	2.42 (1.71–3.41)	1.80 (1.55–2.10)	4.60 (3.97–5.32)	2.38 (1.74–3.23)	5.54 (4.12–7.45)
Age ≥ 65	1.31 (1.23–1.40)	2.60 (2.41–2.81)	1.04 (0.90–1.19)	1.26 (1.04–1.53)	1.40 (1.20–1.64)	2.33 (1.96–2.78)	1.88 (1.78–1.98)	3.24 (3.07–3.42)	2.00 (1.77–2.26)	4.18 (3.71–4.72)
P interaction	0.7992	<0.0001	0.3017	0.0109	0.0053	0.9686	0.5910	<0.0001	0.4433	0.0140
**Sex**										
Female	1.42 (1.30–1.54)	2.38 (2.15–2.62)	0.89 (0.72–1.09)	1.23 (0.96–1.58)	1.78 (1.45–2.20)	2.88 (2.29–3.63)	2.16 (2.00–2.33)	3.70 (3.43–3.99)	2.56 (2.2–2.98)	3.90 (3.33–4.56)
Male	1.24 (1.14–1.34)	2.32 (2.11–2.55)	1.06 (0.97–1.16)	1.35 (1.21–1.5)	1.43 (1.20–1.70)	1.83 (1.47–2.27)	1.70 (1.59–1.82)	3.03 (2.83–3.25)	1.57 (1.32–1.86)	4.72 (4.03–5.53)
P interaction	0.0285	0.7315	0.0858	0.6618	0.0502	0.0001	<0.0001	0.0019	<0.0001	0.3975
**CHA_2_DS_2_-VASc Score**										
CHA_2_DS_2_-VASc Score ≤ 3	1.24 (1.13–1.35)	2.31 (2.09–2.56)	0.95 (0.86–1.05)	1.3 (1.16–1.46)	2.22 (1.85–2.67)	2.56 (2.04–3.22)	1.79 (1.66–1.93)	3.39 (3.13–3.67)	2.24 (1.86–2.7)	5.16 (4.31–6.19)
CHA_2_DS_2_-VASc Score > 3	1.37 (1.27–1.48)	2.42 (2.21–2.65)	0.97 (0.89–1.06)	1.19 (1.06–1.33)	1.08 (0.88–1.32)	2.00 (1.61–2.48)	1.97 (1.84–2.10)	3.31 (3.09–3.53)	1.91 (1.65–2.20)	3.92 (3.40–4.51)
P interaction	0.1575	0.6662	0.8629	0.0209	<0.0001	0.1530	0.1592	0.6183	0.1147	0.0075

CKD, chronic kidney disease. aHR was calculated from adjustment for all variables in Table 1.

**Table 4:** Sensitivity analyses.

	Heart Failure		Stroke or Systemic Thromboembolism		Acute Myocardial Infarction		All-Cause Mortality		Cardiovascular Mortality	
	aHR (95% CI)	*p*-Value	aHR (95% CI)	*p*-Value	aHR (95% CI)	*p*-Value	aHR (95% CI)	*p*-Value	aHR (95% CI)	*p*-Value
**Excluding AF patients with valvular heart disease or hyperthyroidism**										
Non-CKD	1		1		1		1		1	
Prevalent CKD	1.40 (1.31–1.50)	<0.0001	1.05 (0.98–1.13)	0.1606	1.36 (1.16–1.59)	0.0002	1.96 (1.85–2.07)	<0.0001	2.08 (1.83–2.38)	<0.0001
Incident CKD	2.06 (1.90–2.24)	<0.0001	1.20 (1.10–1.32)	<0.0001	2.57 (2.16–3.06)	<0.0001	3.16 (2.98–3.34)	<0.0001	3.51 (3.07–4.02)	<0.0001
**CKD diagnosis according to ICD-9 codes 585**										
Non-CKD	1									
Prevalent CKD	1.36 (1.28–1.45)	<0.0001	1.09 (1.02–1.17)	0.010	1.29 (1.11–1.50)	0.001	1.22 (1.17–1.28)	<0.0001	1.24 (1.11–1.39)	0.0001
Incident CKD	2.51 (2.24–2.82)	<0.0001	1.29 (1.13–1.48)	0.0002	2.91 (2.3–3.68)	<0.0001	2.22 (2.06–2.39)	<0.0001	2.46 (2.08–2.90)	<0.0001
**Adjusted for propensity score**										
Non-CKD	1		1		1		1		1	
Prevalent CKD	1.54 (1.35–1.76)	<0.0001	1.02 (0.88–1.19)	0.7739	1.55 (1.13–2.13)	0.0063	2.41 (2.15–2.69)	<0.0001	2.74 (2.14–3.51)	<0.0001
Incident CKD	2.23 (1.88–2.65)	<0.0001	1.23 (1.02–1.48)	0.0319	1.90 (1.29–2.80)	0.0012	3.50 (3.09–3.96)	<0.0001	4.54 (3.46–5.96)	<0.0001
**Only those with follow-up of more than 1 year**										
Non-CKD	1		1		1		1		1	
Prevalent CKD	1.41 (1.31–1.52)	<0.0001	1.01 (0.94–1.09)	0.7707	1.57 (1.34–1.84)	<0.0001	1.81 (1.72–1.91)	<0.0001	2.07 (1.83–2.34)	<0.0001
Incident CKD	2.03 (1.87–2.20)	<0.0001	1.16 (1.06–1.27)	0.001	2.03 (1.71–2.41)	<0.0001	2.99 (2.83–3.16)	<0.0001	3.56 (3.15–4.02)	<0.0001

Adjusted for all variables in Table 1 with medications (antiplatelet and anticoagulant agents) treated as time-dependent variables. aHR, adjusted hazard ratio; CI, confidence interval; AF, atrial fibrillation; CKD, chronic kidney disease.

## Supplementary Materials

The following are available online at https://www.mdpi.com/2077-0383/8/8/1184/s1, Figure S1: Cumulative survival curves free of hospitalization for heart failure between prevalent CKD, non-CKD and incident CKD groups. Figure S2: Cumulative survival curves free of acute myocardial infarction between prevalent CKD, non-CKD and incident CKD groups. Figure S3: Cumulative survival curves free of stroke or systemic thromboembolism between prevalent CKD, non-CKD and incident CKD groups. Figure S4: Cumulative survival curves free of all-cause death between prevalent CKD, non-CKD and incident CKD groups. Figure S5: Cumulative survival curves free of cardiovascular death between prevalent CKD, non-CKD and incident CKD groups. Table S1: ICD-9-CM codes used to identify chronic kidney disease, atrial fibrillation, comorbidities and the cause of death. Table S2: Risks for heart failure, stroke or systemic thromboembolism, acute myocardial infarction and mortality among patients with AF by CKD status after re-classification of incident CKD as prevalent CKD (non-CKD as the reference). Table S3: Risks for heart failure, stroke or systemic thromboembolism, acute myocardial infarction and mortality among patients with AF by CKD status after re-classification of incident CKD as prevalent CKD (prevalent CKD as the reference).

## References

[1] Sarnak M.J., Levey A.S., Schoolwerth A.C., Coresh J., Culleton B., Hamm L.L., Parfrey P., Klag M.J., Wilson P.W., Raij L. (2003). Kidney disease as a risk factor for development of cardiovascular disease: A statement from the American Heart Association Councils on Kidney in Cardiovascular Disease, High Blood Pressure Research, Clinical Cardiology, and Epidemiology and Prevention. Circulation.

[2] Go A.S., Chertow G.M., Fan D., McCulloch C.E., Hsu C.Y. (2004). Chronic kidney disease and the risks of death, cardiovascular events, and hospitalization. N. Engl. J Med..

[3] Coresh J., Selvin E., Stevens L.A., Manzi J., Kusek J.W., Eggers P., Levey A.S., Lente F.V. (2007). Prevalence of chronic kidney disease in the United States. JAMA.

[4] Pálsson R., Patel U.D. (2014). Cardiovascular complications of diabetic kidney disease. Adv. Chronic Kidney Dis..

[5] Go A.S., Hylek E.M., Phillips K.A., Chang Y., Henault L.E., Selby J.V., Singer D.E. (2001). Prevalence of diagnosed atrial fibrillation in adults: National implications for rhythm management and stroke prevention: The AnTicoagulation and Risk Factors in Atrial Fibrillation (ATRIA) study. JAMA.

[6] Chugh S.S., Havmoeller R., Narayanan K., Singh D., Rienstra M., Benjamin E.J., Forouzanfar M.H., Naghavi M., Mensah G.A., Ezzati M. (2014). Worldwide epidemiology of atrial fibrillation: A Global Burden of Disease 2010 Study. Circulation.

[7] Schnabel R.B., Rienstra M., Sullivan L.M., Sun J.X., Moser C.B., Levy D., Lubitz S.A., Wang T.J., Ellinor P.T., Tadros T.M. (2013). Risk assessment for incident heart failure in individuals with atrial fibrillation. Eur. J Heart Fail.

[8] Soliman E.Z., Lopez F., O’Neal W.T., Chen L.Y., Bengtson L., Zhang Z.M., Alonso A., Loehr L., Cushman M. (2015). Atrial fibrillation and risk of ST-segment-elevation versus non-ST-segment-elevation myocardial infarction: The Atherosclerosis Risk in Communities (ARIC) study. Circulation.

[9] Alonso A., Lopez F.L., Matsushita K., Loehr L.R., Agarwal S.K., Chen L.Y., Coresh J., Soliman E.Z., Astor B.C. (2011). Chronic kidney disease is associated with the incidence of atrial fibrillation: The Atherosclerosis Risk in Communities (ARIC) study. Circulation.

[10] Bansal N., Xie D., Tao K., Chen J., Deo R., Horwitz E., Raj D., Kallem R.K., Keane M.G., Lora C.M. (2016). CRIC Study. Atrial Fibrillation and Risk of ESRD in Adults with CKD. Clin. J. Am. Soc. Nephrol..

[11] Ananthapanyasut W., Napan S., Rudolph E.H., Harindhanavudhi T., Ayash H., Guglielmi K.E., Lerma E.V. (2010). Prevalence of atrial fibrillation and its predictors in nondialysis patients with chronic kidney disease. Clin. J. Am. Soc. Nephrol..

[12] Odutayo A., Wong C.X., Hsiao A.J., Hopewell S., Altman D.G., Emdin C.A. (2016). Atrial fibrillation and risks of cardiovascular disease, renal disease, and death Systematic review and meta-analysis. BMJ.

[13] Hsu T.W., Liu J.S., Hung S.C., Kuo K.L., Chang Y.K., Chen Y.C., Tarng D.C., Hsu C.C. (2014). Renoprotective effect of renin-angiotensin-aldosterone system blockade in patients with predialysis advanced chronic kidney disease, hypertension, and anemia. JAMA Intern. Med..

[14] Wu C.Y., Chen Y.J., Ho H.J., Hsu Y.C., Kuo K.N., Wu M.S., Lin J.T. (2012). Association between nucleoside analogues and risk of hepatitis B virus-related hepatocellular carcinoma recurrence following liver resection. JAMA.

[15] January C.T., Wann L.S., Alpert J.S., Calkins H., Cigarroa J.E., Cleveland J.C., Murray K.T., Conti J.B., Ellinor P.T., Ezekowitz M.D. (2014). 2014 AHA/ACC/HRS guideline for the management of patients with atrial fibrillation: A report of the American College of Cardiology/American Heart Association Task Force on Practice Guidelines and the Heart Rhythm Society. J. Am. Coll. Cardiol..

[16] McCaffrey D.F., Griffin B.A., Almirall D., Slaughter M.E., Ramchand R., Burgette L.F. (2013). A tutorial on propensity score estimation for multiple treatments using generalized boosted models. Stat. Med..

[17] Hsu P.K., Kor C.T., Hsieh Y.P. (2018). Effect of New-Onset Diabetes Mellitus on Renal Outcomes and Mortality in Patients with Chronic Kidney Disease. J. Clin. Med..

[18] Go A.S., Fang M.C., Udaltsova N., Chang Y., Pomernacki N.K., Borowsky L. (2009). ATRIA Study Investigators. Impact of proteinuria and glomerular filtration rate on risk of thromboembolism in atrial fibrillation: The anticoagulation and risk factors in atrial fibrillation (ATRIA) study. Circulation.

[19] Hohnloser S.H., Hijazi Z., Thomas L., Alexander J.H., Amerena J., Hanna M., Granger C.B., Keltai M., Lanas F., Lopes R.D. (2012). Efficacy of apixaban when compared with warfarin in relation to renal function in patients with atrial fibrillation: Insights from the ARISTOTLE trial. Eur. Heart J..

[20] Hohnloser S.H., Oldgren J., Yang S., Wallentin L., Ezekowitz M., Reilly P., Connolly S.J., Eikelboom J., Brueckmann M., Yusuf S. (2012). Myocardial ischemic events in patients with atrial fibrillation treated with dabigatran or warfarin in the RE-LY (Randomized Evaluation of Long-Term Anticoagulation Therapy) trial. Circulation.

[21] Nakagawa K., Hirai T., Takashima S., Fukuda N., Ohara K., Sasahara E., Inoue H., Taguchi Y., Dougu N., Nozawa T. (2011). Chronic kidney disease and CHADS(2) score independently predict cardiovascular events and mortality in patients with nonvalvular atrial fibrillation. Am. J. Cardiol..

[22] Piccini J.P., Stevens S.R., Chang Y., Singer D.E., Lokhnygina Y., Go A.S., Patel M.R., Mahaffey K.W., Halperin J.L., Breithardt G. (2013). Renal dysfunction as a predictor of stroke and systemic embolism in patients with nonvalvular atrial fibrillation: Validation of the R2CHADS2 Index in the ROCKET AF and ATRIA Study Cohorts. Circulation.

[23] Guo Y., Wang H., Zhao X., Zhang Y., Zhang D., Ma J., Lip G.Y., Wang Y. (2013). Sequential changes in renal function and the risk of stroke and death in patients with atrial fibrillation. Int. J. Cardiol..

[24] Uhlig K., Levey A.S., Sarnak M.J. (2003). Traditional cardiac risk factors in individuals with chronic kidney disease. Semin. Dial..

[25] Madore F. (2003). Uremia-related metabolic cardiac risk factors in chronic kidney disease. Semin. Dial..

[26] Himmelfarb J., Stenvinkel P., Ikizler T.A., Hakim R.M. (2002). The elephant in uremia: Oxidant stress as a unifying concept of cardiovascular disease in uremia. Kidney Int..

[27] Annuk M., Soveri I., Zilmer M., Lind L., Hulthe J., Fellström B. (2005). Endothelial function, CRP and oxidative stress in chronic kidney disease. J. Nephrol..

[28] Lau W.L., Huisa B.N., Fisher M. (2017). The Cerebrovascular-Chronic Kidney Disease Connection: Perspectives and Mechanisms. Transl. Stroke Res..

[29] Heine G.H., Gerhart M.K., Ulrich C., Kaler H., Girndt M. (2005). Renal Doppler resistance indices are associated with systemic atherosclerosis in kidney transplant recipients. Kidney Int..

[30] Zhang F., Liu H., Liu D., Liu Y., Li H., Tan X., Zhang H., Liu F., Peng Y. (2017). Effects of RAAS Inhibitors in Patients with Kidney Disease. Curr. Hypertens. Rep..

[31] Khatib R., Joseph P., Briel M., Yusuf S., Healey J. (2013). Blockade of the renin-angiotensin-aldosterone system (RAAS) for primary prevention of non-valvular atrial fibrillation: A systematic review and meta analysis of randomized controlled trials. Int. J. Cardiol..

[32] Lin M.Y., Chiu Y.W., Chang J.S., Lin H.L., Lee C.T.C., Chiu G.F., Hwang S.J., Kuo M.C., Wu M.T., Chen H.C. (2015). Association of prescribed Chinese herbal medicine use with risk of end-stage renal disease in patients with chronic kidney disease. Kidney Int..

[33] Weng S.C., Wu C.L., Kor C.T., Chiu P.F., Wu M.J., Chang C.C., Tarng D.C. (2017). Migraine and subsequent chronic kidney disease risk: A nationwide population-based cohort study. BMJ Open.

[34] Chen Y.C., Su Y.C., Li C.Y., Wu C.P., Lee M.S. (2015). A nationwide cohort study suggests chronic hepatitis B virus infection increases the risk of end-stage renal disease among patients in Taiwan. Kidney Int..

