# Supplementary material for: Effects of Prevalent and Incident Chronic Kidney Disease on Cardiovascular Events in Patients with Atrial Fibrillation
Source: J Clin Med. 2019 Aug 7;8(8):1184. doi: 10.3390/jcm8081184 (PMC6723547; doi:10.3390/jcm8081184)
Supplement: Supplementary file 1 [file jcm-08-01184-s001.pdf]

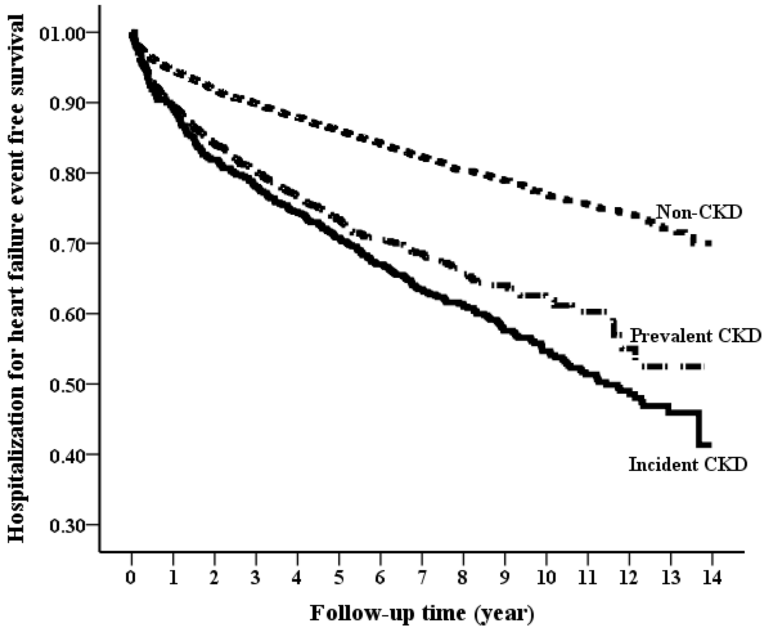

Figure S1. Cumulative survival curves free of hospitalization for heart failure between prevalent CKD, non-CKD and incident CKD groups.

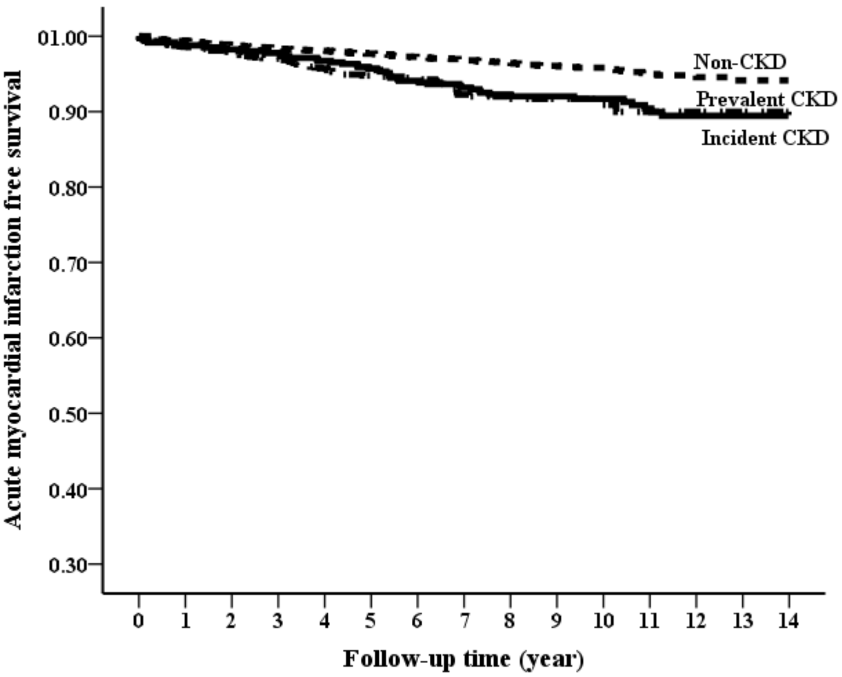

Figure S2. Cumulative survival curves free of acute myocardial infarction between prevalent CKD, non-CKD and incident CKD groups.

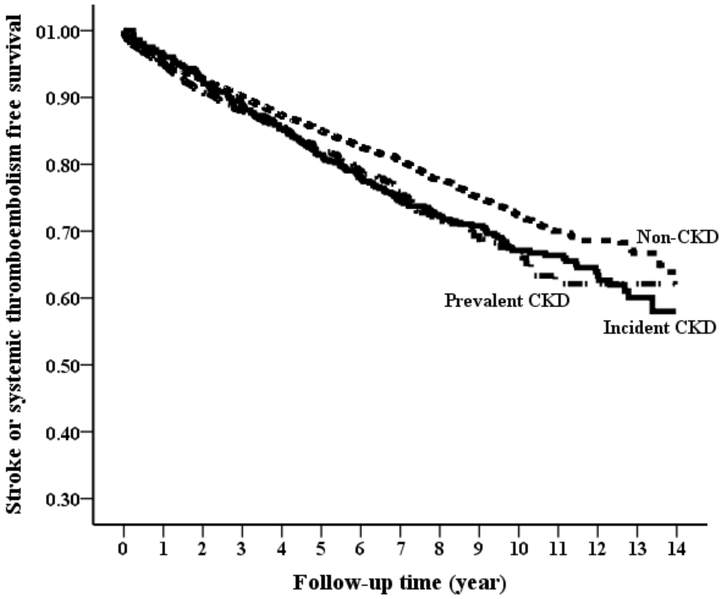

Figure S3. Cumulative survival curves free of stroke or systemic thromboembolism between prevalent CKD, non-CKD and incident CKD groups.

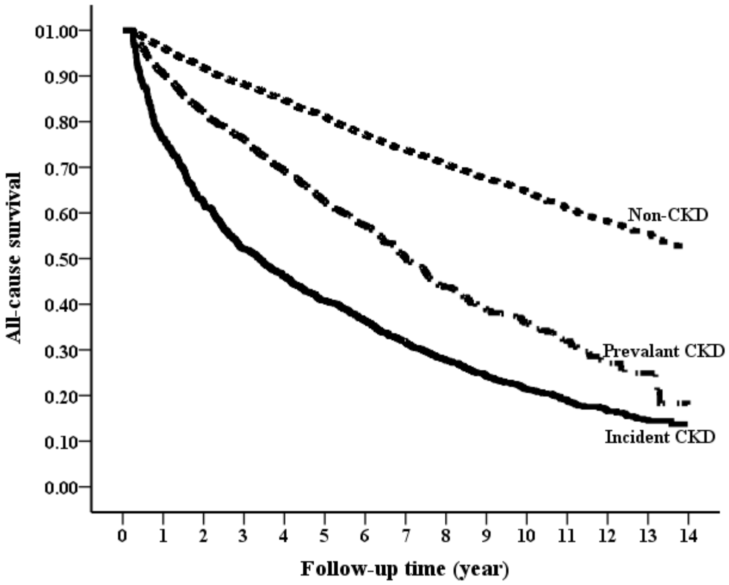

Figure S4. Cumulative survival curves free of all-cause death between prevalent CKD, non-CKD and incident CKD groups.

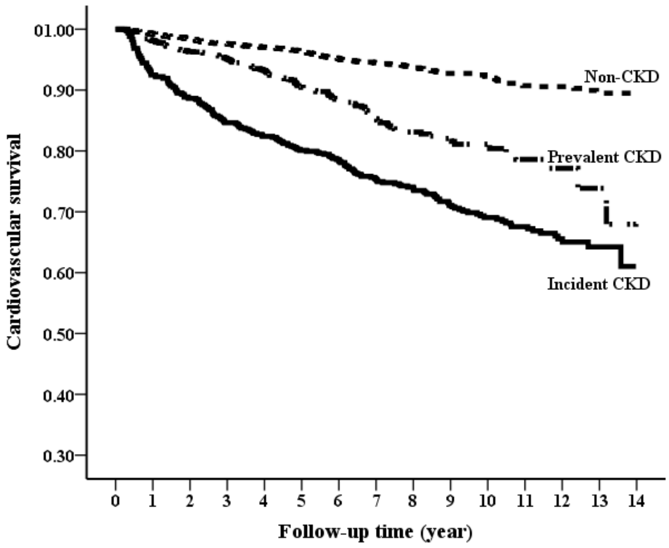

Figure S5. Cumulative survival curves free of cardiovascular death between prevalent CKD, non-CKD and incident CKD groups.

Table S1. ICD-9-CM codes used to identify chronic kidney disease, atrial fibrillation, comorbidities and the cause of death.

**Diseases**	**Corresponding ICD-9-CM codes**
Chronic kidney disease	580-589
Atrial fibrillation	427.31
Acute myocardial infarction	410, 429.5, 429.6, 429.71, 429.79
Co-morbid diseases	
Ischemic heart disease	410.x–414.x
COPD	491.x, 492.x, 496.x
Cancer	140-165, 170-172, 174-195, 200-208
Liver cirrhosis	571
Dementia	290
Rheumatoid disease	446.5, 710, 714, 725
CHA_2DS_2-VASc	
Heart failure	428.x
Hypertension	401.x–405.x
Diabetes mellitus	250.x
Ischemic stroke	433, 434
Transient ischemic attack	435.9, 435.2, 435.8
Peripheral artery embolism	444
Pulmonary embolism	415.0, 415.9
Acute myocardial infarction	410, 429.5, 429.6, 429.71, 429.79
Aortic plaque	440.0, 440.2, 440.30, 440.31, 440.32, 440.71, 440.8, 440.9, 441, 443.9, 443.91
**Causes of Death**	
Cardiovascular death	390.x – 398.x, 410.x – 414.x, 4151, 41511, 41519, 420.x, 422.x – 429.x, 431.x, 433.x – 436.x, 518.4, 785.51

Abbreviation: COPD, chronic obstructive pulmonary disease; ICD-9-CM, International Classification of Disease, 9th Revision, Clinical Modification.

Table S2. Risks for heart failure, stroke or systemic thromboembolism, acute myocardial infarction and mortality among patients with AF by CKD status after re-classification of incident CKD as prevalent CKD (non-CKD as the reference).

	Within 30 days		Within 90 days		Within 180 days	
	**aHR (95% CI)**	**P-value**	**aHR (95% CI)**	**P-value**	**aHR (95% CI)**	**P-value**
**Heart failure**						
Non-CKD	1		1		1	
Prevalent CKD	1.39(1.31–1.47)	<0.0001	1.44(1.36-1.52)	<0.0001	1.56(1.47–1.64)	<0.0001
Incident CKD	2.39(2.22–2.56)	<0.0001	2.48(2.3–2.67)	<0.0001	2.34(2.16–2.53)	<0.0001
**Stroke or systemic thromboembolism**						
Non–CKD	1		1		1	
Prevalent CKD	0.98(0.92–1.05)	0.5725	0.99(0.93–1.06)	0.8385	1(0.94–1.07)	0.9021
Incident CKD	1.24(1.14–1.34)	<0.0001	1.20(1.10–1.31)	<0.0001	1.23(1.12–1.34)	<0.0001
**Acute myocardial infarction**						
Non–CKD	1		1		1	
Prevalent CKD	1.58(1.38–1.8)	<0.0001	1.52(1.34–1.74)	<0.0001	1.63(1.43–1.86)	<0.0001
Incident CKD	2.4(2.04–2.81)	<0.0001	2.55(2.16–3.00)	<0.0001	2.87(2.42–3.39)	<0.0001
**All-cause mortality**						
Non-CKD	1		1		1	
Prevalent CKD	1.87(1.78–1.96)	<0.0001	1.90(1.81–2.00)	<0.0001	1.95(1.86–2.04)	<0.0001
Incident CKD	3.51(3.34–3.70)	<0.0001	3.65(3.46–3.85)	<0.0001	3.72(3.52–3.93)	<0.0001
**Cardiovascular death**						
Non-CKD	1		1		1	
Prevalent CKD	2.03(1.82–2.27)	<0.0001	2.05(1.84–2.29)	<0.0001	2.2(1.98–2.45)	<0.0001
Incident CKD	4.45(3.97–4.98)	<0.0001	4.74(4.23–5.32)	<0.0001	4.58(4.07–5.17)	<0.0001

CI, confidence interval; HR, hazard ratio; CKD, chronic kidney disease; AF, atrial fibrillation. aHR was calculated from adjustment for all variables in Table 1.

Table S3. Risks for heart failure, stroke or systemic thromboembolism, acute myocardial infarction and mortality among patients with AF by CKD status after re-classification of incident CKD as prevalent CKD (prevalent CKD as the reference).

	Within 30 days		Within 90 days		Within 180 days	
	**aHR (95% CI)**	**P-value**	**aHR (95% CI)**	**P-value**	**aHR (95% CI)**	**P-value**
**Heart failure**						
Non-CKD	0.72(0.68–0.76)	<0.0001	0.69(0.66–0.73)	<0.0001	0.64(0.61–0.68)	<0.0001
Prevalent CKD	1		1		1	

Incident CKD	1.72(1.6–1.85)	<0.0001	1.72(1.59–1.86)	<0.0001	1.5(1.39–1.63)	<0.0001
**Stroke or systemic thromboembolism**						
Non-CKD	1.02(0.96–1.09)	0.5725	1.01(0.95–1.07)	0.8385	1.00(0.94–1.06)	0.9021
Prevalent CKD	1		1		1	
Incident CKD	1.26(1.15–1.38)	<0.0001	1.21(1.1–1.33)	<0.0001	1.22(1.11–1.34)	<0.0001
**Acute myocardial infarction**						
Non-CKD	0.63(0.56–0.72)	<0.0001	0.66(0.58–0.75)	<0.0001	0.61(0.54–0.7)	<0.0001
Prevalent CKD	1		1		1	
Incident CKD	1.52(1.29–1.78)	<0.0001	1.67(1.42–1.97)	<0.0001	1.76(1.49–2.07)	<0.0001
**All-cause mortality**						
Non-CKD	0.54(0.51–0.56)	<0.0001	0.53(0.5–0.55)	<0.0001	0.51(0.49–0.54)	<0.0001
Prevalent CKD	1		1		1	
Incident CKD	1.88(1.79–1.98)	<0.0001	1.92(1.82–2.02)	<0.0001	1.91(1.82–2.01)	<0.0001
**Cardiovascular death**						
Non-CKD	0.49(0.44–0.55)	<0.0001	0.49(0.44–0.54)	<0.0001	0.45(0.41–0.50)	<0.0001
Prevalent CKD	1		1		1	
Incident CKD	2.19(1.98–2.44)	<0.0001	2.29(2.06–2.55)	<0.0001	2.05(1.84–2.28)	<0.0001

CI, confidence interval; HR, hazard ratio; CKD, chronic kidney disease; AF, atrial fibrillation. aHR was calculated from adjustment for all variables in Table 1.
